# Epidemiological patterns of new psychoactive substances use in Tunisian school adolescents, 2021

**DOI:** 10.1192/j.eurpsy.2023.1123

**Published:** 2023-07-19

**Authors:** A. Silini, S. Rejaibi, M. Zid, I. Ben Slema, R. Mallekh, N. Zoghlami, S. Ben Youssef, M. Zribi, N. Ben Salah, H. Aounallah-Skhiri

**Affiliations:** 1 Department of Epidemiology, National Institute of Health; 2 Nutrition Surveillance and Epidemiology department, SURVEN Research Laboratory; 3 Medical Faculty of Medicine, Tunis El Manar University; 4 Intensive Care Unit department, Center for Urgent Medical Assistance, Tunis, Tunisia

## Abstract

**Introduction:**

The emergence of New Psycho-active Substances (NPS) such as Synthetic cannabinoid and cathinone, represents a challenging issue for drug policy globally. In order to set up new adjusted measures to limit this phenomenon extension, objective epidemiologic indicators are requested.

**Objectives:**

We aimed to determine the prevalence of Synthetic cannabinoid and cathinone consumption in Tunisian adolescents by gender and regional distribution.

**Methods:**

Data from the Mediterranean school survey on alcohol and other drugs (MedSPAD III-2021) were used. Based on three-stage stratification sampling method, high school teenagers in first and second grades of secondary education, were enrolled. Data collection was performed using a self-administered standardized questionnaire. We examined weighted prevalence estimates of NPS use at least once in a lifetime (Synthetic cannabinoid and cathinone) by gender and regional distribution. Epi data software was used for data entry and all statistical analysis were performed with STATA software.

**Results:**

The survey included 6201 adolescents with a mean age of 16.8 years and a sex ratio female/male of 1.5. Synthetic cannabinoid’s use was reported by 1.9%, 95% CI [1.57-2.39] of students, with statistically significant difference between boys (4.1%) and girls (0.6%), p-value<10^-4^ . This consumption was the highest in Tunis the capital city, the center-east and the north-east (2.7%, 2.2% and 2% respectively). As for synthetic cathinone’s use, it was reported by 0.36% 95%CI [0.24-0.56] of our study sample, with statistically significant difference between boys (0.8%) and girls (0.8%), p-value<10^-4^ .

**Conclusions:**

Our study highlighted an emerging use of NPS among high school students with significant male predominance. Further research on NPS epidemiology is, hence, needed to reinforce evidence-based management strategies aiming at fighting this phenomenon. Sensitization of decision makers to control accessibility, and increasing awareness among adolescents’ close family / school-staff environment regarding this issue, are strongly recommended.

**Disclosure of Interest:**

None Declared

